# Subadditive responses to extremely short blue and green pulsed light on visual evoked potentials, pupillary constriction and electroretinograms

**DOI:** 10.1186/s40101-017-0156-4

**Published:** 2017-11-17

**Authors:** Soomin Lee, Yuria Uchiyama, Yoshihiro Shimomura, Tetsuo Katsuura

**Affiliations:** 1 Center for Environment, Health and Sciences, Chiba University, 6-2-1 Kashiwanoha, Kashiwa, Chiba 277-0882 Japan; 2 Graduate School of Engineering, Chiba University, Chiba, Japan

## Abstract

**Background:**

The simultaneous exposure to blue and green light was reported to result in less melatonin suppression than monochromatic exposure to blue or green light. Here, we conducted an experiment using extremely short blue- and green-pulsed light to examine their visual and nonvisual effects on visual evoked potentials (VEPs), pupillary constriction, electroretinograms (ERGs), and subjective evaluations.

**Methods:**

Twelve adult male subjects were exposed to three light conditions: blue-pulsed light (2.5-ms pulse width), green-pulsed light (2.5-ms pulse width), and simultaneous blue- and green-pulsed light with white background light. We measured the subject’s pupil diameter three times in each condition. Then, after 10 min of rest, the subject was exposed to the same three light conditions. We measured the averaged ERG and VEP during 210 pulsed-light exposures in each condition. We also determined subjective evaluations using a visual analog scale (VAS) method.

**Results:**

The pupillary constriction during the simultaneous exposure to blue- and green-pulsed light was significantly lower than that during the blue-pulsed light exposure despite the double irradiance intensity of the combination. We also found that the b/|a| wave of the ERGs during the simultaneous exposure to blue- and green-pulsed light was lower than that during the blue-pulsed light exposure. We confirmed the subadditive response to pulsed light on pupillary constriction and ERG. However, the P100 of the VEPs during the blue-pulsed light were smaller than those during the simultaneous blue- and green-pulsed light and green-pulsed light, indicating that the P100 amplitude might depend on the luminance of light.

**Conclusions:**

Our findings demonstrated the effect of the subadditive response to extremely short pulsed light on pupillary constriction and ERG responses. The effects on ipRGCs by the blue-pulsed light exposure are apparently reduced by the simultaneous irradiation of green light. The blue versus yellow (b/y) bipolar cells in the retina might be responsible for this phenomenon.

## Introduction

Intrinsically photosensitive retinal ganglion cells (ipRGCs) affect the suprachiasmatic nucleus and act as the primary photoreceptor for nonvisual effects such as the suppression of pineal melatonin synthesis [1–3], circadian photoentrainment [4–6], and other behavioral and physiological functions [5–8]. The discovery of ipRGCs that express the photopigment melanopsin and send retinal information to the brain centers for controlling pupil size has led to a better understanding of retinal mechanisms contributing to pupil responses [4, 7, 9–20]. The ipRGCs respond to short-wavelength light of around 480 nm [21–23], and the contribution of ipRGCs to the pupillary light response was greater at a higher irradiance, with the role of the rods being more dominant at a lower irradiance level [4, 15, 17, 24]. Besides, we found that the pupillary response under the extremely short (pulse width 100 μs) but higher irradiance (11.2 μW/cm^2^ or 13.4 log photons/cm^2^/s) blue-pulsed light was significantly greater than that under the steady blue light (1.4 μW/cm^2^ or 12.5 log photons/cm^2^/s) which had equal blue light components (the products of irradiance and duration) [12]. We assumed that the pulsed light of strong irradiance might induce significantly greater pupillary response [25].

By the way, Figueiro et al. [26] found that simultaneous exposure to blue and green light resulted in less melatonin suppression than monochromatic light exposure to blue or green light. This effect was named the “subadditive response” to light [26]. We defined the “subadditive response to light” as the effect of simultaneous exposure to several color lights was smaller than that of monochromatic light exposure. In our previous study, we observed that pupillary constriction during the simultaneous exposure to pulses of blue and green light was smaller than during exposure to a pulse of blue light despite the double irradiance intensity of the combination [27]. Subadditive responses were also found in other studies of monochromatic and polychromatic light on melatonin suppression [28–30]. By contrast, Papamichael et al. [31] examined the effect of the concurrent administration of blue and red monochromatic light on acute melatonin suppression and subjective mood and alertness responses in humans. They reported that the primary determinant of the melatonin suppression response was the irradiance of 479 nm blue light, and this was unaffected by a simultaneous administration of red light [31]. In this way, a subadditive response for the spectral power distribution of the light has not yet been confirmed.

Moreover, it remains unclear whether the subadditive response affects pupillary constriction. An electroretinogram (ERG) has been used in a few studies to inspect ipRGC function [32, 33], commonly recorded by using electrodes such as a contact lens that contacted the cornea. However, these types of electrodes have the potential to cause corneal abrasions and infections of the cornea [34]. ERGs have alternatively been recorded by using skin electrodes [34–42]. These studies suggested that ERG responses of skin electrodes had a smaller amplitude but were quite similar to those of eye-contact electrodes [37, 38, 41, 42]. Recently, ERG responses have been successfully measured by using the skin electrodes with a pulse reference power line noise reduction (PURE) method [39, 40] to assess the retinal functions, demonstrating the utility of this approach.

Taking advantage of this recent advancement in ERG technology, this study comprehensively clarified the visual and nonvisual effects of blue and green light in order to understand the subadditive response to blue light more fully. In this study, we measured visual evoked potentials (VEPs), pupillary constriction, ERGs, and subjective evaluations in response to extremely short pulsed blue and green light.

## Methods

### Subjects

Twelve healthy young males (age 24 ± 1.3 years, body height 168.6 ± 4.1 cm, body weight 59.2 ± 7.7 kg) with normal color vision participated in the experiment. We confirmed that the subjects had normal color vision by administering the Farnsworth Munsell 100 Hue Test. The subjects were sufficiently informed about the experimental procedure and gave informed consent. They were not allowed to perform vigorous exercise or to consume alcoholic drinks or caffeine-containing foods before the experiment. This experiment was approved by the Ethics Committee of the Graduate School of Engineering, Chiba University (No. 26-08).

### Lighting condition

**Fig. 1:**
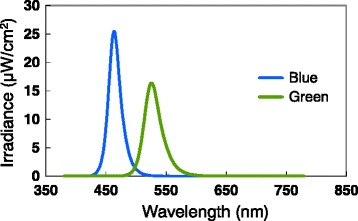
Spectral irradiance of light

Each subject was exposed simultaneously or separately to 2.5 ms of blue and/or green extremely short pulsed lights at 1.6 × 10^15^ photons/cm^2^/s or 15.2 log photons/cm^2^/s (Table 1) with white background light from an incandescent lamp (2287 K, 30 cd/m^2^), using an integrating sphere (Takano Co., Nagano, Japan) in a booth (1420 × 510 × 1750 mm). The spectral distribution for the green and blue light-emitting diodes (LEDs) and the incandescent lamp was measured at each subject’s eye level using a spectroradiometer (CL-500A, Konica Minolta Optics Co., Tokyo). The wavelength of peak emission from the blue LED was 464 nm and that from the green LED was 526 nm (Fig. 1). The full width at half maximum of these LEDs was 22 nm and 31 nm, respectively (Table 1). The experiment was conducted in a climatic chamber (TBR-6HA4G2C; Espec Corp., Osaka, Japan) in which the air temperature and relative humidity were set at 25 °C and 50%, respectively.

**Table 1:** Characteristics of the light conditions

Light condition	Blue	Green	Blue + Green
Peak wavelength (nm)	464	526	–
FWHM (nm)	22	31	–
Irradiance (μW/cm^2^)	686	607	1294
Photon density (10^15^ photons/cm^2^/s)	1.61	1.62	3.24
Photon density (log photons/cm^2^/s)	15.2	15.2	15.5
Photopic illuminance (lx)	439	3281	3734
Scotopic illuminance (lx)	7388	7836	15,204
Melanopsin-stimulating irradiance (*μ*W/cm^2^)	526.1	343.8	868.6
Melanopsin-stimulating photon density (10^14^ photons/cm^2^/s)	13.0	8.5	21.5

*FWHM* full width at half maximum

### Procedure and measurements

**Fig. 2:**
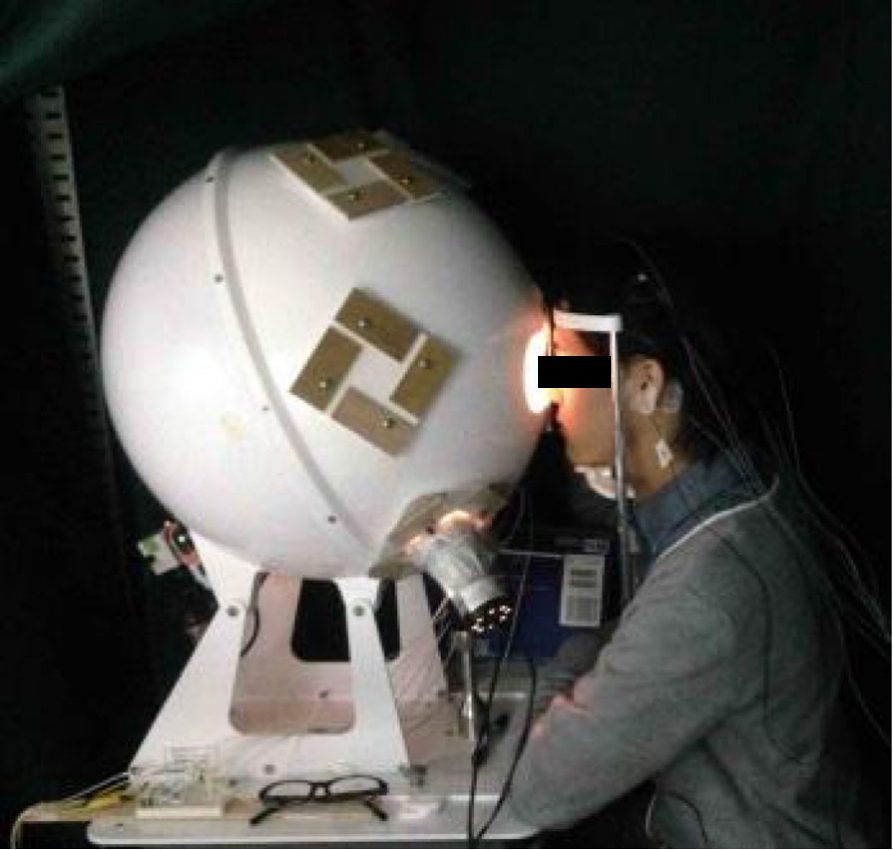
Integrating sphere and a subject

The subject sat on a chair with his eyes facing the integrating sphere (Fig. 2). Blue and green LEDs and an incandescent lamp were arrayed in the integrating sphere. After 10 min of base light exposure (2287 K, 30 cd/m^2^), the subject was exposed to three pulsed lights of blue (Blue), green (Green), or simultaneous blue and green (Blue + Green) light every 10 s with a white background light for measuring the pupil diameter (EMR-8B, nac Image Technology, Tokyo, Japan). From the measurement of the pupil diameter (PD), we calculated the pupillary constriction ratio (% pupillary constriction) as follows: % pupillary constriction = [(baseline PD − minimum PD after light exposure)/baseline PD] × 100, and we defined the time to 75% recovery of the pupil diameter to the baseline as the recovery time.

**Fig. 3:**
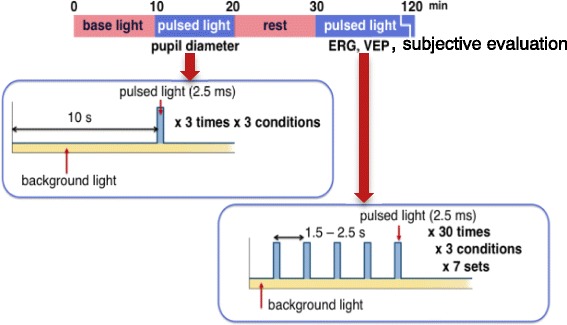
The procedure of the experiment

Next, after 10 min of rest in the baseline light condition, the subject’s left eye was exposed to the same three light conditions at random intervals (1.5–2.5 s) during 90 min. We measured the averaged ERG and VEP during 210 pulsed-light exposures in each condition. We also determined subjective evaluations of “bluish” and “greenish” using a visual analog scale (VAS) method after each condition (Fig. 3).

**Fig. 4:**
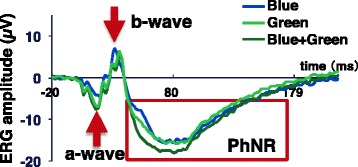
Grand average of ERG

We obtained averaged ERGs using an evoked response recorder (PuREC, Mayo Co., Aichi, Japan) with a pulse reference power line noise reduction (PURE) method [39, 40] and skin electrodes from 90 artifact-free ERG signals. The active skin electrode was placed on the orbital rim 7 mm from the margin of the lower eyelid of the left eye, and the reference electrode was placed on the same position on the right eye. The earth electrode was attached to the left earlobe. ERG signals were digitized at 1250 Hz and amplified with a digital band-pass filter of 0.3–200 Hz. From the averaged ERG, we analyzed the amplitude of a wave, b wave, and photopic negative responses (PhNRs) (Fig. 4). The a wave amplitude was calculated as the maximum value of 10–50 ms after the light exposure minus the baseline average. The b wave amplitude was measured from trough of the a wave to the peak of the b wave located in 20–60 ms after the light exposure. The b/|a| was obtained as the b wave amplitude divided by the absolute value of the a wave (|a|). The PhNRmax was then calculated as the maximum negative value of 40–200 ms after the light exposure minus the baseline average.

VEP waveforms were averaged from at least 180 artifact-free electroencephalogram (EEG) recordings at Fz, C3, Cz, C4, O1, and O2 electrode sites. EEG signals were digitized at 1000 Hz and amplified with digital band-pass filter of 1.0–100 Hz (Biopac Systems, Goleta, CA, USA).

The order of the three light conditions was counterbalanced among the subjects.

### Statistical analysis

We used a one-way repeated measures analysis of variance (ANOVA) (PASW Statistic 18; Ver. 18.0J, IBM, Armonk, NY, USA) to evaluate the effects of the light factor except in the VEPs. In the VEPs, a two-way repeated-measures ANOVA (light factor × region factor) was conducted. When any significant effect was found, multiple comparisons of the light condition were performed by the Bonferroni method. Probability (*p*) values < 0.05 were accepted as significant.

## Results

**Fig. 5:**
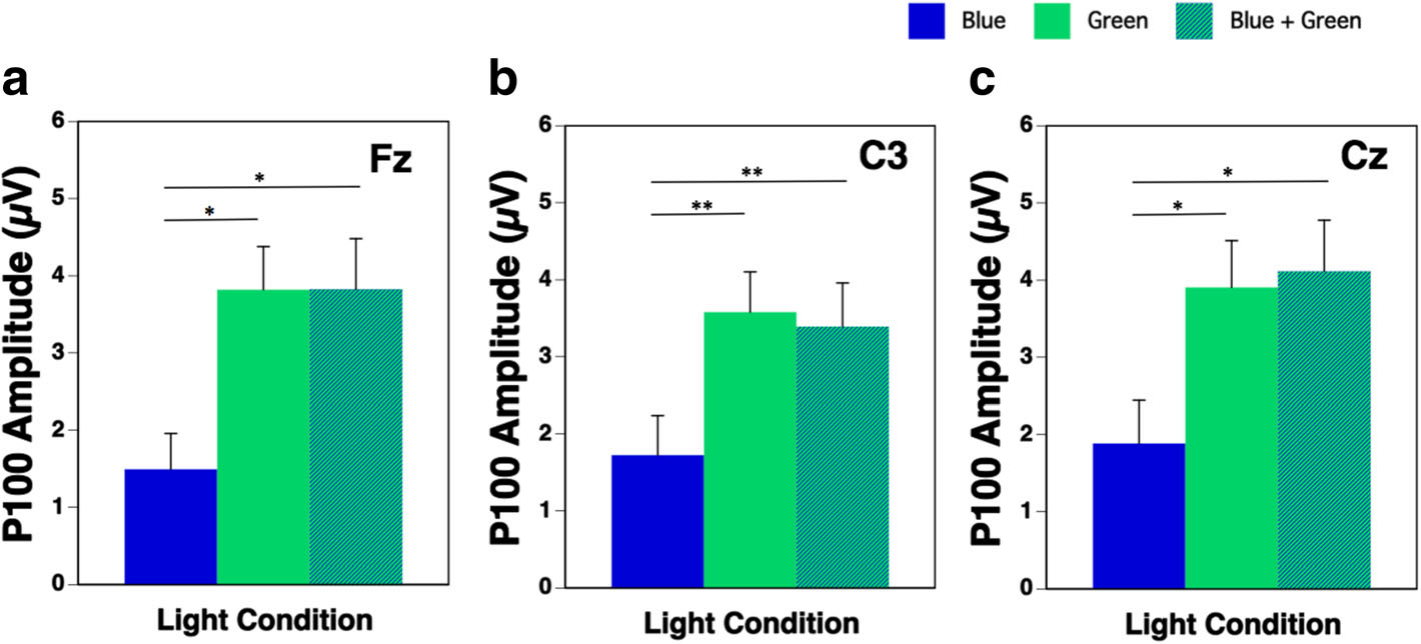
P100 amplitude at Fz (**a**), C3 (**b**), and Cz (**c**) during the three light conditions (Blue: blue-pulsed light; green: green-pulsed light; Blue + Green: simultaneous blue- and green-pulsed light; *p < 0.05; **p < 0.01)

Two-way repeated measures ANOVA on P100 amplitude of VEPs revealed that the main effects of the light condition and the electrode site were significant, and the interaction effect was also significant. Therefore, a one-way repeated measures ANOVA was applied to evaluate the effects of the light condition on the P100 amplitude at each region of electrode sites. We found that the main effects of light on the P100 amplitude at the Fz, C3, Cz, and C4 electrode sites were significant (all *p* < 0.01). Multiple comparisons using the Bonferroni procedure found that the P100 amplitudes at the Fz, C3, and Cz electrode sites during the Blue condition were significantly smaller than those during the Blue + Green condition, and those at the same electrode sites during the Blue condition were also significantly smaller than those during Green condition (Fig. 5a–c; *p* < 0.05 or *p* < 0.01). There were no significant main effects of the light condition on the P100 amplitude at the Pz, O1, and O2 sites.

**Fig. 6:**
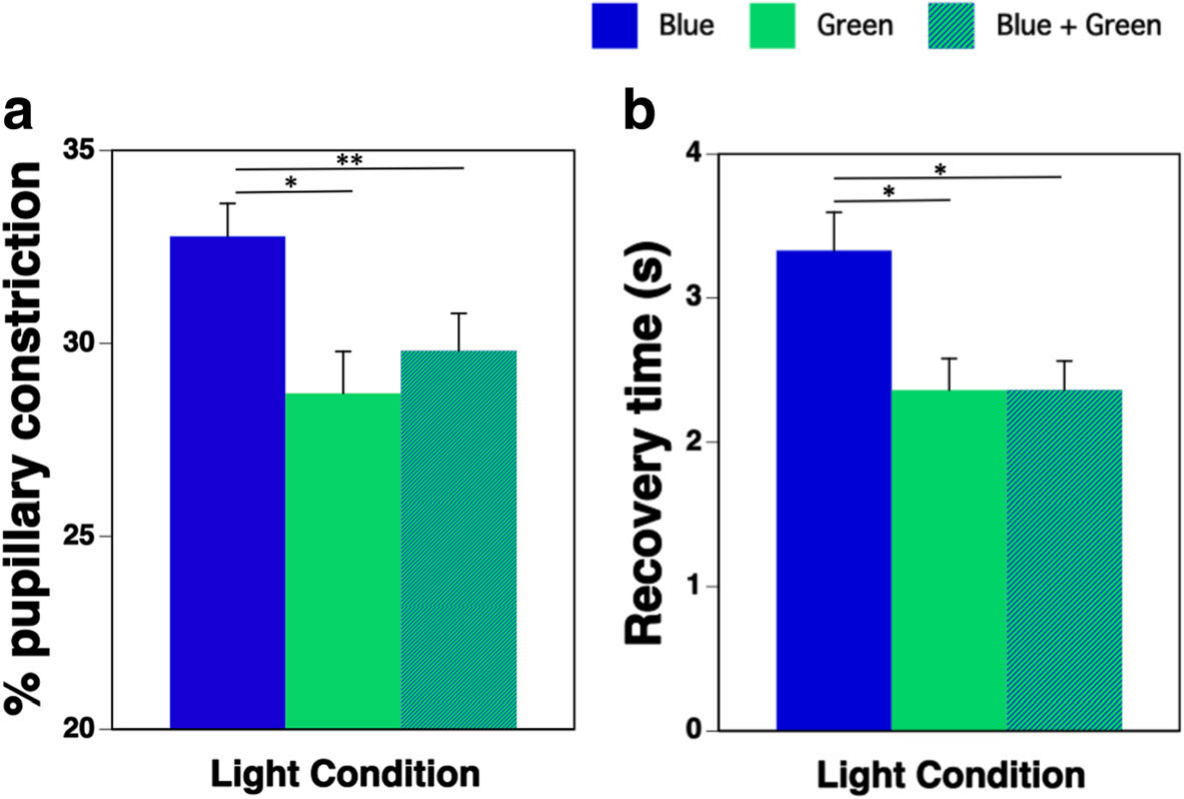
Percentage of pupillary constriction (**a**) and recovery time (**b**) during the three light conditions (B: blue-pulsed light, G: green-pulsed light, B + G: simultaneous blue- and green-pulsed light;* *p* < 0.05, **p < 0.01)

We found that the main effects of light on the percentage of pupillary constriction and the recovery time were significant (both *p* < 0.01). Multiple comparisons using the Bonferroni procedure revealed that the percentage of pupillary constriction during the Blue + Green condition was significantly lower than that during the Blue condition (Fig. 6a; *p* < 0.01), and the recovery time during the Blue + Green condition was significantly shorter than that during the Blue condition (Fig. 6b; *p* < 0.05) despite the double irradiance intensity of the combination.

**Fig. 7:**
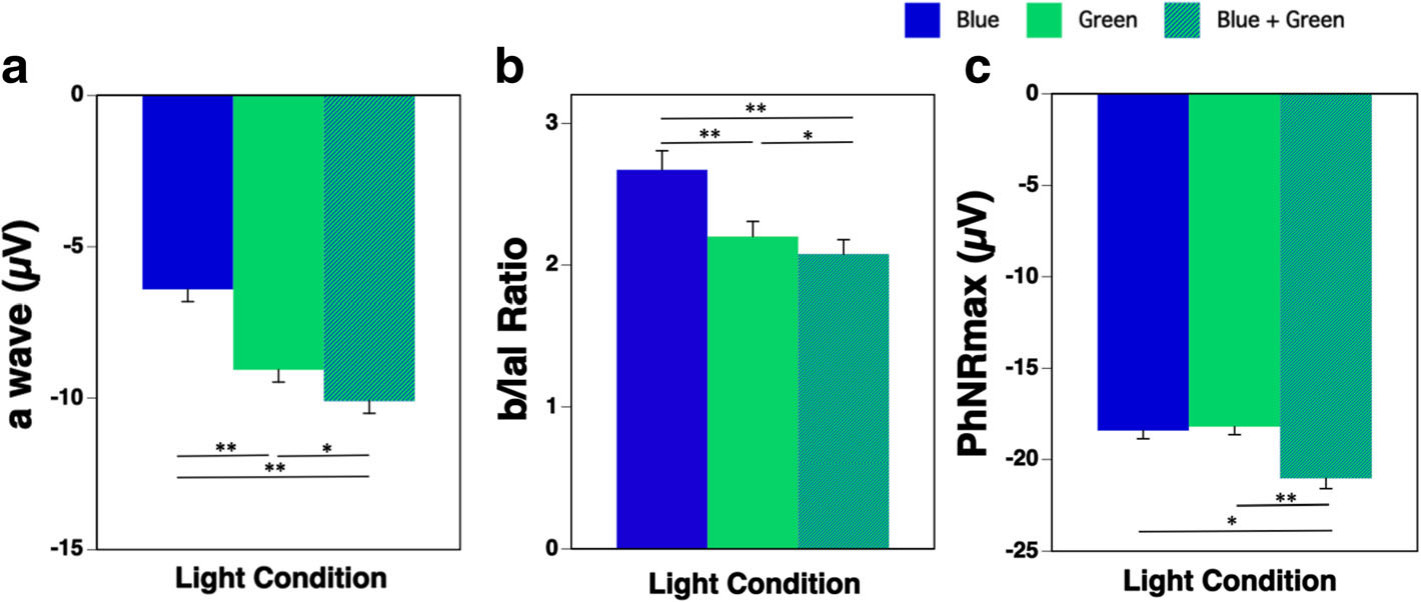
ERG components of a wave (**a**), b/|a| ratio (**b**), and PhNRmax (**c**) during the three light conditions (Blue: blue-pulsed light; Green: green-pulsed light; Blue + Green: simultaneous blue- and green-pulsed light; *p < 0.05; **p < 0.01)

Figure 7 shows the results of the ERG components (a wave, b/|a|, and PhNRmax) during the three light conditions. One-way repeated measures ANOVA on a wave, b/|a|, and PhNRmax showed a significant (all *p* < 0.01) main effect of light. The a wave during the Blue condition was significantly smaller than that during the Blue + Green condition (Fig. 7a; *p* < 0.01). The b/|a| of the ERG during the Blue + Green condition resulted in a significantly smaller ratio than that during the Blue or Green condition (Fig. 7b; *p* < 0.01 and *p* < 0.05, respectively). In addition, the PhNRmax during the Blue + Green condition was significantly larger than that during the Blue or Green condition (Fig. 7c; *p* < 0.05 and *p* < 0.01, respectively).

**Fig. 8:**
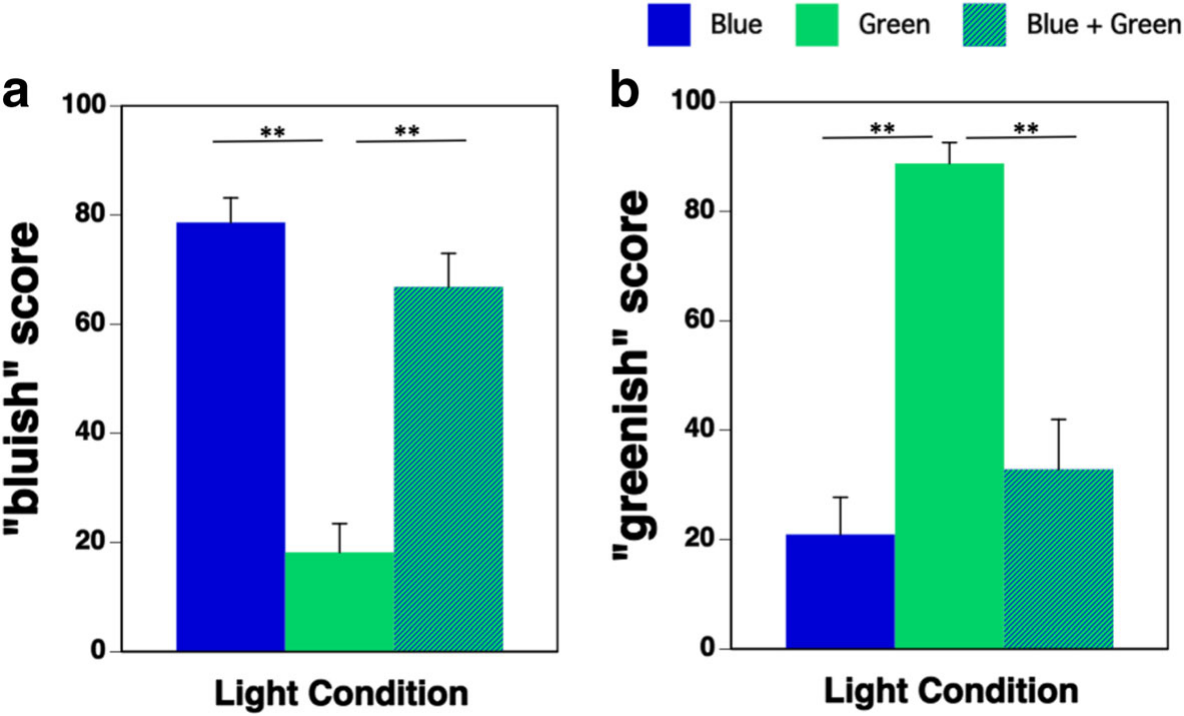
“Bluish” (**a**) and “greenish” (**b**) scores during the three light conditions (Blue: blue-pulsed light; Green: green-pulsed light; Blue + Green: simultaneous blue- and green-pulsed light; **p < 0.01)

There were no significant differences in the results of the subjective evaluations of “bluish” and “greenish” scores between the Blue and the Blue + Green condition (Fig. 8).

## Discussion

Our findings demonstrated a subadditive response to extremely short pulsed light in terms of pupillary constriction and ERG responses, but no subadditive response was evident in the VEP results. We discussed in each of these results in turn below and interpreted what they might mean in terms of the cell types in the retina that might be responsible for this phenomenon.

### VEPs convey the visual, but not the nonvisual, response to blue and green light

The P100 amplitude of the VEPs at the Fz, Cz, and C3 electrode sites during the Blue + Green condition was significantly higher than that during the Blue condition, but there were no significant differences in the P100 amplitudes of the VEPs at these sites during the Green and Blue + Green conditions. There was no subadditive response in the VEPs. Paulus et al. [43] reported that the P100 reflected the external stimuli volume response and increased with the increase in luminance. In the present study, the luminance differed among the three lighting conditions (Blue 245 cd/m^2^, Green 2043 cd/m^2^, and Blue + Green 2250 cd/m^2^) because the photon density of the blue and green light was standardized as 1.6 × 10^15^ photons/cm^2^/s. We thus presumed that the P100 response did not reflect the nonvisual system but rather reflected the visual system including the luminance. There was no subadditive response in the VEPs. This result suggested that the activation of the brain depended on the luminance and reflects the brightness—i.e., the visual system was activated—at around 100 ms after the light stimuli presentation.

### Pupillary constriction shows a subadditive response to blue and green light

We observed that the percentage of pupillary constriction during the Blue condition was greater than that during the Green condition. This result was in agreement with the previous studies [7, 27]. The recovery time during the Blue condition was longer than that during the Green condition. Other studies have demonstrated that the ipRGCs respond to short-wavelength light of around 480 nm [21–23]. The ipRGCs have been reported to contribute to the pupillary response to light in mice at an irradiance level greater than about 13 log photons/cm^2^/s of 470 nm light at the eye level [17]. In the present study, the irradiance intensity of Blue was 15.2 log photons/cm^2^/s, which was much higher than the threshold intensity for activation of ipRGCs. The ipRGCs were shown to contribute to pupillary constriction as a nonvisual effect [15], and they reacted slowly and longwise after the light stimulation [22]. In fact, the result of the pupillary constriction and the recovery time did not reflect the luminance difference among the three lighting conditions. Therefore, we propose that the increase in pupillary constriction and the delay in recovery time during the Blue condition reflect the ipRGCs’ action. The findings of the current experiment show that the nonvisual effect of blue light exposure was prominent in pupillary constriction and the recovery time.

The most important finding in the present study was that the percentage of pupillary constriction in response to the Blue + Green condition was more inhibited and shortened compared to that during the Blue condition, despite the double irradiance intensity of the combination. This showed an apparent subadditive response to pulsed light.

The information of the light wavelength is processed by bipolar cells, horizontal cells, and amacrine cells after being outputted from three types of cone [44], and this information is converted into red/green (r/g), blue/yellow (b/y), and brightness information in the ganglion cells, before traveling on to the visual cortex via the optic nerves. The spectral opponent b/y bipolar cells have been hypothesized to provide direct input to the ipRGCs [26]. The ipRGCs show an unusual “color-opponent” receptive field in which an S-cone-mediated OFF response is antagonistic to an (L + M) cone-mediated ON response in the in vitro primate retina [22], and an S-cone-mediated ON response is opposed to an (L + M) cone-mediated OFF response [45]. Therefore, the responses of ipRGCs activated by S-cones might be reduced by inhibition from (L + M) cones on simultaneous exposure to blue and green light.

### A subadditive response to blue and green light is evident in the ERG

To assess the retinal functions more directly, we also measured ERG in the present study. ERG response has commonly several components of a and b waves [46, 47]. Although their precise origin and meaning remain to be elucidated [32], previous studies indicated that the a wave shows an initial response after light exposure and reflects the response of photoreceptors, especially cone cells [48, 49], and pharmacological studies revealed that the b wave reflects ON bipolar activities in the retina [46, 47].

The a wave amplitude of the ERGs during the present Blue + Green condition was significantly larger than that during the Blue and Green condition, respectively. In addition, the a wave amplitude during the Green condition was greater than that during the Blue condition. There was no subadditive response in the a wave component of the ERGs. This result might be related to the difference in the luminance (blue 245 cd/m^2^, green 2043 cd/m^2^) even though the photon density of the Blue condition was equal to that of the Green condition, because the a wave mainly reflects the response of cone cells [48, 49].

The b wave amplitude depends on the a wave and the integrity of signal transmission within the retina; therefore, the b/|a| depends only on retinal function [50]. The b/|a| values during the Blue and Green conditions were larger than those in the Blue + Green condition. These results confirmed that the subadditive response occurred in the retina. Thus, we speculate that in the present study, the subadditive response occurred along the process of the transmission of the information from bipolar cells to ipRGCs within the retina.

The output from bipolar cells is then transmitted to ipRGCs, and the result of the photoreceptor output appears in a slow negative potential (PhNRs) following the a and b waves of photopic ERG, as was found in animal studies [51–54] and in a human study [42]. The PhNR has been reported to originate from the spiking activity of the ipRGCs and their axons, with contributions from amacrine and surrounding glial cells in the retina [51–54]. We confirmed that the PhNRmax during the Blue + Green condition was significantly greater than that during the Blue and Green conditions; therefore, it was not difficult to estimate the additive response in the PhNR.

There were no significant differences in the subjective evaluations of the “bluish” and “greenish” scores between the Blue and Blue + Green condition. This showed that the effect of the simultaneous irradiation of Green on the perception of color was very small.

## Conclusions

We examined VEPs, pupillary constriction, and ERGs during separate and simultaneous exposure to extremely short pulses of blue and green light. We found that the P100 amplitude of the VEP during the simultaneous exposure to pulses of blue and green light was significantly higher than that during the blue light exposure. We concluded that the P100 response did not reflect the nonvisual system but rather reflected the visual system including the luminance. However, pupillary constriction during exposure to a blue light was significantly greater than during exposure to a green light. Interestingly, pupillary constriction during the simultaneous exposure to blue and green light was smaller than during exposure to a blue light despite the double irradiance intensity of the combination. In addition, the b/|a| values of ERG during the blue and green light exposures were larger than those in the Blue + Green exposure. Our findings demonstrated the effect of the subadditive response to extremely short pulsed light on pupillary constriction and the ERG as a nonvisual effect. The responses to the blue-pulsed light exposure were apparently reduced by the simultaneous irradiation of green-pulsed light. The blue versus yellow (b/y) bipolar cells in the retina might be responsible for this phenomenon.

## Abbreviations

ipRGCs: Intrinsically photosensitive retinal ganglion cells
LED: Light-emitting diode
